# Malignant transformation of hepatocellular adenoma: report of a case

**DOI:** 10.11604/pamj.2020.35.92.17416

**Published:** 2020-03-26

**Authors:** Mohamed Mouhoub, Achraf Miry, Anass Haloui, Nassira Karich, Imane Kamaoui, Mehdi Soufi, Amal Bennani

**Affiliations:** 1 Pathology Department, Mohamed VI University Hospital, Oujda, Morocco; 2 Radiology Department, Mohamed VI University Hospital, Oujda, Morocco; 3 General Surgery Department, Mohamed VI University Hospital, Oujda, Morocco

**Keywords:** Hepatocellular adenoma, hepatocellular carcinoma, malignant transformation

## Abstract

Hepatocellular adenomas are benign liver tumours that occur mainly but not exclusively in young women taking contraceptives. Their malignant transformation into hepatocellular carcinoma is a rare complication that has been rarely reported in women taking contraceptives. The purpose of our work is to remind the epidemiological and diagnostic features of malignant transformation of hepatic adenomas by reporting the case of a hepatocellular carcinoma developed from a hepatocellular adenoma diagnosed within the Pathology Department of the Mohammed VI university hospital of Oujda.

## Introduction

Hepatocellular adenomas often affect young women of childbearing age and rarely occur in males or children [1]. Patients most often present for a fortuitous abdominal mass discovered incidentally when performing an imaging examination for another indication. The malignant transformation of hepatocellular adenomas into hepatocellular carcinoma is a rare complication occurring in of 4.5 to 9% of all hepatocellular adenomas [2, 3]. Patients having glycogen storage disease, a history of taking androgen or anabolic steroids, male patients and those having hepatocellular adenomas by beta-catenin activation are at higher risk of malignant transformation to hepatocellular carcinoma [2, 4, 5]. We report the case of a hepatocellular carcinoma developed from a hepatocellular adenoma with a discussion about the epidemiological and diagnostic features of the malignant transformation of hepatocellular adenoma.

## Patient and observation

**Figure 1:**
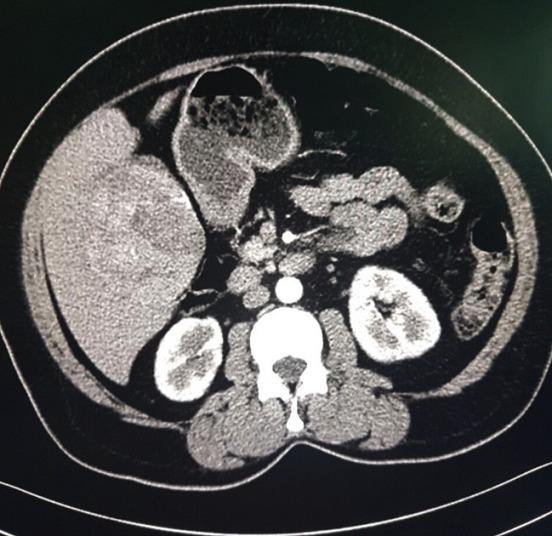
Abdominal CT scan showing a hepatic tissular mass that displays an arterial contrast enhancement

**Figure 2:**
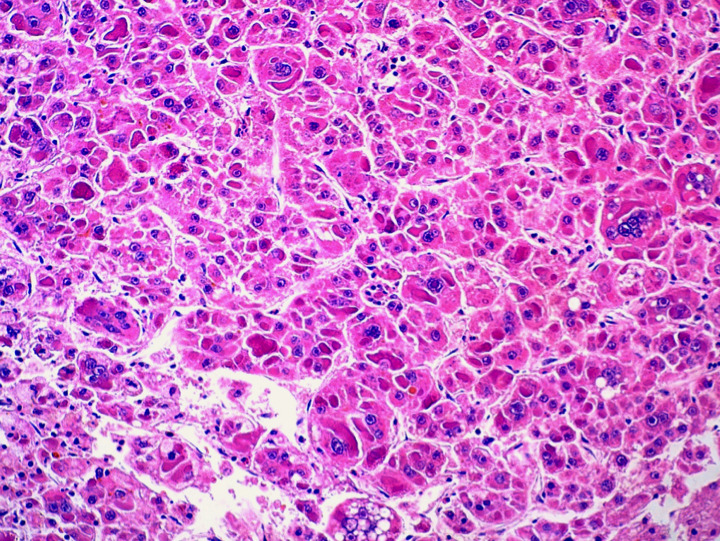
Microphotography showing a compact growth pattern with hepatocytes having enlarged atypical nuclei and displaying numerous mitotic figures

**Figure 3:**
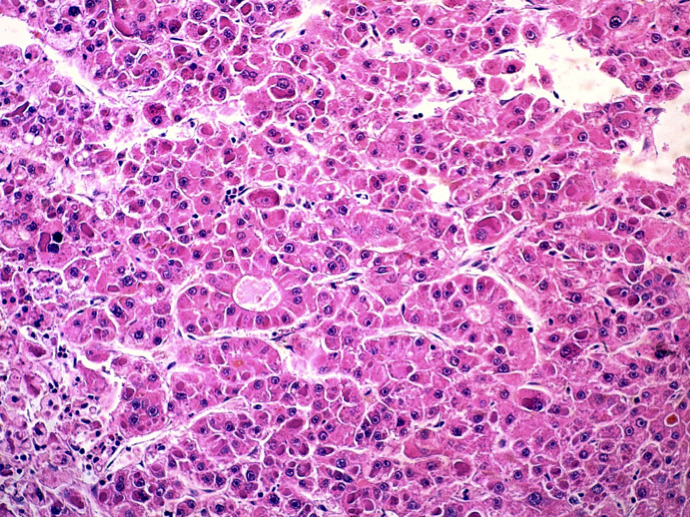
Microphotography showing a pseudo glandular growth pattern

**Figure 4:**
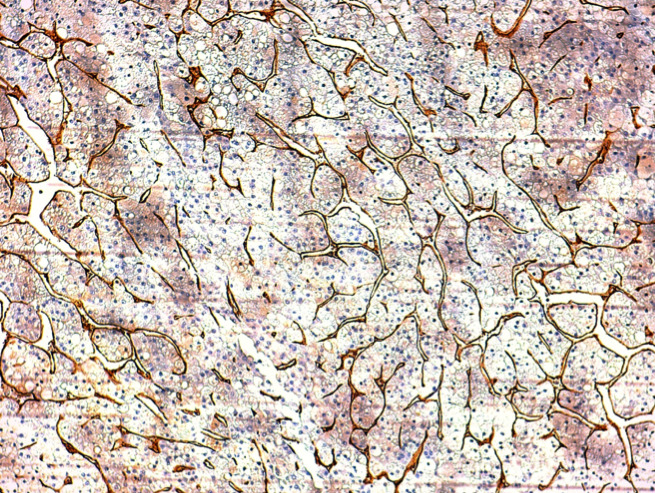
Microphotography showing that sinusoid endothelial cells are stained by anti-CD34 antibodies

This is the case of a 57-year-old woman, 6 gravida 6 para, who presented with 4 months history of right hypochondrium pain that was associated with diarrhoea without any notion of weight loss. She had a history of diabetes mellitus for 14 years. Her body mass index is 28kg/m^2^. An axial abdominal CT scan was performed and revealed the presence of an 8 x 6 x 4cm hypodense mass of irregular contours involving the IV, V and VI segments. This mass is tissular and displays an arterial and portal contrast enhancement (Figure 1). Delayed mass washouts with enhancement lasting in the tumour capsule were observed. Pathological examination revealed a well-circumscribed capsulated tumour. The fibrous capsule gave rise to few fibrous septas containing scattered inflammatory mononuclear cells. The proliferation is made of 1 to 2 hepatocytes thick trabeculae. The cells have eosinophilic or micro and macro vesicular optically clear cytoplasmic droplets. Nuclei are regular and finely nucleated. Focally, nodules of compact pattern are distinguished, with hepatocytes having enlarged atypical nuclei and displaying numerous mitotic figures. Hyaline cytoplasmic globules are conspicuous (Figure 2). The compact pattern was associated to more focal pseudo glandular and trabecular patterns (Figure 3). Atypical pleomorphic giant multinucleated hepatocytes were also present. The sinusoids seemed to be focally capillarized, which has been confirmed by the positivity to the anti-CD34 (Figure 4).

## Discussion

The malignant transformation of hepatocellular adenomas into hepatocellular carcinoma is a rare complication that occurs in 4.5 to 9% of cases [2, 3]. Patients having a history of a glycogen storage disease, taking androgen or anabolic steroids, male patients and hepatocellular adenoma cases secondary to the beta-catenin pathway activation are at higher risk of malignant transformation to hepatocellular carcinoma [2, 4, 5]. However, the malignant transformation of hepatocellular adenoma has also been reported though rarely in women taking contraceptives without evidence of mutations in beta-catenin neither immunohistochemically nor molecularly [6]. The risk of malignant transformation also appears high when tumour size exceeds 4cm. Histologically, a malignant transformation of hepatocellular adenoma can be demonstrated by the presence of foci discreetly or frankly malignant within the hepatocellular adenoma. Hepatocellular carcinoma foci typically have a loss of the reticulin framework, cells displaying a high nuclear-cytoplasmic ratio, a variable level of nuclear pleomorphism and numerous mitotic figures [7]. Malignant hepatocytes may exhibit trabecular, pseudo-glandular or solid pattern of proliferation. Positive GPC3 labelling, a membrane heparan-sulfate proteoglycan, has a diagnostic value when other elements are in favour of a hepatocellular carcinoma [8]. Hepatocellular adenoma lesions, focal nodular hyperplasia and regenerative nodules are all negative for GPC3. Typical hepatocellular carcinoma is also positive for CD34 that stains the endothelial cells of the tumour sinusoids though early lesions have a very focal staining. Hepatocellular adenoma may also sometimes have diffuse CD34 staining at the level of sinusoidal endothelial cells. Further research is needed to identify new markers that can predict the development of hepatocellular carcinoma on hepatocellular adenoma to help manage high-risk patients.

## Conclusion

Hepatocellular adenomas are benign tumours of the liver that occur mainly but not exclusively in young women taking contraceptives. Hepatocellular adenomas are due to the proliferation of hepatocytes containing high levels of glycogen and fat but whose architecture is abnormal. The malignant transformation of hepatocellular adenomas into hepatocellular carcinoma is a rare complication. We report the case of a hepatocellular carcinoma developed from hepatocellular adenoma with a literature review of the epidemiological and diagnostic features of the malignant transformation of hepatocellular adenoma.

## Competing interests

The authors declare no competing interests.
